# Cannabidiol reverses depression-like behaviors by enhancing hippocampal synaptic plasticity in rats with chronic restraint stress

**DOI:** 10.14202/vetworld.2025.2823-2838

**Published:** 2025-09-23

**Authors:** Jutamas Ruanpang, Namphung Thongta, Anchalee Vattarakorn, Sukonthar Ngampramuan, Pornjira Pariwatcharakul, Sompol Tapechum, Chit Care, Narawut Pakaprot

**Affiliations:** 1 Department of Physiology, Faculty of Medicine Siriraj Hospital, Mahidol University, Bangkok, 10700, Thailand; 2 Research Center for Neuroscience, Institute of Molecular Biosciences, Mahidol University, Nakhon Pathom, 73170, Thailand; 3 Department of Psychiatry, Faculty of Medicine Siriraj Hospital, Mahidol University, Bangkok, 10700, Thailand

**Keywords:** cannabidiol, chronic stress, depression, hippocampus, long-term potentiation, synaptic plasticity

## Abstract

**Background and Aim:**

Major depressive disorder is a prevalent psychiatric condition associated with impaired neuroplasticity, particularly in the hippocampus. Although selective serotonin reuptake inhibitors (SSRIs) are commonly prescribed, their delayed onset and adverse effects highlight the need for alternative therapies. Cannabidiol (CBD), a non-psychotomimetic cannabinoid, has shown antidepressant-like properties, but its mechanistic link to hippocampal synaptic plasticity remains unclear. This study aimed to evaluate the effects of CBD on depression-like behaviors and hippocampal neuroplasticity in rats subjected to chronic restraint stress (CRS).

**Materials and Methods:**

Sixty male Wistar rats were randomly divided into six groups: Non-stressed vehicle (NV), CRS vehicle (SV), escitalopram-treated CRS (SE, 10 mg/kg), and CBD-treated CRS at 10, 30, or 100 mg/kg (SC10, SC30, and SC100). Rats were subjected to CRS for 28 days and treated daily through intraperitoneal injection. Depression-like behaviors were assessed using the forced swim test (FST) and sucrose preference test (SPT). Locomotor activity was evaluated through the open-field test (OFT). Hippocampal dendritic spine density (Golgi–Cox staining) and long-term potentiation (LTP, electrophysiology) were measured on day 28.

**Results:**

CRS induced behavioral despair (↑ immobility in FST) and anhedonia (↓ sucrose preference in SPT), accompanied by reduced hippocampal spine density. At all doses, CBD significantly reduced immobility, comparable to escitalopram. Notably, only CBD at 100 mg/kg and escitalopram reversed anhedonia. All CBD-treated groups showed an increase in dendritic spine density, with SC10 producing the greatest enhancement. Moreover, CBD at 100 mg/kg markedly improved hippocampal LTP at 1 h and 2 h post-stimulation, an effect not observed with escitalopram. Locomotor activity remained unaffected.

**Conclusion:**

CBD demonstrated potent antidepressant-like effects in a CRS rat model, alleviating behavioral despair and anhedonia while enhancing hippocampal dendritic spine density and synaptic strength. These findings suggest CBD as a promising candidate for stress-related mood disorders, with mechanistic actions distinct from conventional SSRIs and potential utility in patients unresponsive to current therapies.

## INTRODUCTION

Depression is a prevalent and serious mental health disorder that profoundly affects emotions, cognition, and daily functioning. It is characterized by persistent sadness, hopelessness (despair), and anhedonia, the loss of interest or pleasure in previously enjoyable activities, leading to substantial impacts on quality of life and physical health [1]. The pathophysiology underlying depression is multifactorial and remains incompletely understood, involving complex interactions between genetic, biochemical, and neurobiological factors [1]. Among the most recognized mechanisms are neurotransmitter imbalances and impaired neuroplasticity [2, 3]. The monoamine hypothesis continues to be a central theory, positing that reduced levels of monoamine neurotransmitters, including serotonin (5-hydroxytryptamine [5-HT]), norepinephrine (NA), and dopamine, contribute to depressive symptoms [4]. Dysfunction of the serotonergic system, in particular, plays a key role in this condition.

Selective serotonin reuptake inhibitors (SSRIs) are widely prescribed antidepressants that act by blocking the serotonin transporter, thereby elevating extracellular 5-HT levels and alleviating depressive symptoms [5]. In addition to modulating monoamine availability, SSRIs and other antidepressant therapies influence neurotrophic signaling pathways, particularly those involving brain-derived neurotrophic factor (BDNF) [6, 7]. SSRIs enhance serotonergic neurotransmission, which subsequently increases BDNF expression [8]. Elevated serotonin activates postsynaptic receptors, stimulating cyclic adenosine monophosphate (cAMP) signaling, which phosphorylates cAMP response element-binding protein. This transcription factor promotes BDNF expression, thereby supporting synaptic plasticity [9, 10]. Because BDNF and its receptor, tropomyosin receptor kinase B (TrkB), are essential for neuroplasticity, reduced BDNF levels in depression disrupt synaptic remodeling, mood regulation, and cognitive processes [11]. Thus, therapeutic approaches often target the BDNF/TrkB pathway to restore neuroplasticity and mitigate depressive symptoms [9, 10]. Nevertheless, SSRIs are limited by delayed therapeutic onset and adverse effects, underscoring the need for alternative agents with faster action and improved tolerability [12].

Cannabidiol (CBD), a naturally occurring cannabinoid (CB), has emerged as a promising candidate. Unlike tetrahydrocannabinol, CBD lacks psychotomimetic effects and does not produce the marijuana-associated “high” [13–15]. Preclinical and animal studies by Elsaid *et al*. [13] and Reuveni *et al*. [16] suggest that CBD possesses antidepressant potential, though its precise mechanisms remain uncertain. Evidence indicates that CBD may upregulate BDNF–TrkB signaling and activate downstream molecular pathways that enhance synaptic plasticity [17–19]. In addition, CBD exerts diverse pharmacological actions by interacting with multiple receptor systems, producing dose-dependent behavioral and neurobiological effects [20, 21].

Despite the extensive use of SSRIs as first-line treatments for major depressive disorder (MDD), their limitations, including delayed therapeutic onset, variable efficacy, and adverse side effects, restrict their clinical utility [12]. Furthermore, while SSRIs primarily modulate monoaminergic transmission and indirectly enhance BDNF expression, they do not consistently restore synaptic plasticity across all patients [6–8]. Increasing evidence highlights the critical role of hippocampal neuroplasticity, including dendritic spine remodeling and long-term potentiation (LTP), in the pathophysiology of depression and its treatment response [11, 22–24]. However, existing pharmacotherapies fail to adequately target or enhance these mechanisms in a reliable and sustained manner.

CBD, a non-psychotomimetic CB, has attracted growing attention for its potential antidepressant-like effects [13, 16]. Preclinical studies [17–19, 25] suggest that CBD may modulate BDNF–TrkB signaling and endocannabinoid pathways to enhance synaptic plasticity, yet the mechanistic links between CBD’s antidepressant effects and structural or functional hippocampal changes remain poorly defined. While behavioral studies [17, 26, 27] have reported CBD’s efficacy in alleviating depression-like symptoms under chronic stress conditions, few have systematically examined whether CBD can simultaneously reverse depression-related behaviors and promote measurable neuroplastic adaptations, such as increased dendritic spine density or enhanced LTP. Moreover, dose-dependent effects of CBD on hippocampal function remain unclear, with some evidence suggesting that low and high doses may exert distinct or even opposing outcomes [28, 29]. These knowledge gaps limit our understanding of CBD’s therapeutic potential and translational value in depression management.

This study aimed to investigate the antidepressant-like effects of CBD in a rat model of chronic restraint stress (CRS), with a particular focus on its ability to modulate hippocampal synaptic plasticity. Specifically, the objectives were to: (i) evaluate the impact of CBD on depression-like behaviors, including behavioral despair and anhedonia, using the forced swim test (FST) and sucrose preference test (SPT); (ii) assess the effect of CBD on hippocampal dendritic spine density as a structural marker of neuroplasticity; and (iii) examine changes in hippocampal LTP as a functional correlate of synaptic strength. Escitalopram, a standard SSRI, was included as a positive control to enable direct comparison with a clinically established antidepressant. By integrating behavioral, structural, and electrophysiological assessments, this study sought to elucidate the neurobiological mechanisms underlying CBD’s antidepressant-like effects and determine whether CBD offers mechanistic advantages over SSRIs in restoring hippocampal plasticity.

## MATERIALS AND METHODS

### Ethical approval

The Animal Ethics Committee of the Faculty of Medicine Siriraj Hospital, Mahidol University approved this study (Approval number: SI-ACUP 003/2564).

### Study period and location

This study was conducted from August 9, 2021, to May 17, 2024, at the Faculty of Medicine Siriraj Hospital, Mahidol University, Thailand.

### Animals

Sixty male Wistar rats (weight: 180–200 g; age: 7 weeks) purchased from Nomura Siam International, Thailand, were acclimated at the Siriraj Laboratory Animal Research and Care Center until the experiments commenced. The rats were then housed under standard conditions with controlled humidity (40%–70%), a temperature of 25°C, and a 12 h light–dark cycle. Rats were fed with standard chow and water *ad libitum*.

### Experimental design

The rats were randomly allocated into six groups (10 per group):

1. Non-stressed animals treated with a vehicle (non-stressed vehicle [NV])
2. CRS animals treated with a vehicle (CRS vehicle [SV]) as the negative control group
3. CRS animals treated with a 10 mg/kg dose of escitalopram, an SSRI antidepressant (SE), as the positive control group
4. CRS animals treated with a 10 mg/kg dose of CBD (SC10)
5. CRS animals treated with a 30 mg/kg dose of CBD (SC30)
6. CRS animals treated with a 100 mg/kg dose of CBD (SC100).

Rats were coded, listed in an Excel spreadsheet 2016 (Microsoft Office, Washington, USA), and randomly assigned to experimental groups using the randomization function of Excel. The sample size was calculated using G*Power version 3.1 (Heinrich-Heine University of Düsseldorf, Germany). With an effect size of 0.5, a significance level (alpha) of 0.05, and a statistical power of 80%, the minimum required sample size was 10 animals/group.

**Figure 1:**
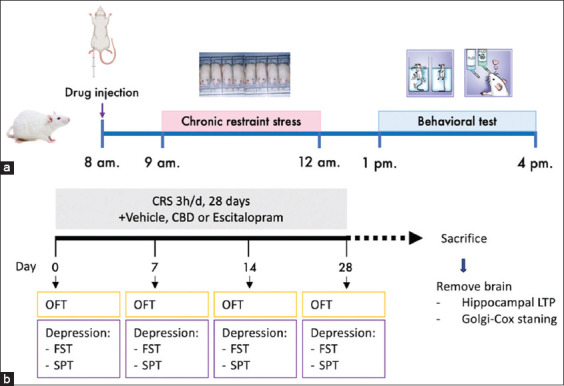
Experimental design. (a) Daily experimental protocol. (b) Experimental protocol and behavioral tests.

The rats were intraperitoneally injected with the vehicle, escitalopram, or CBD 1 h before CRS (Figure 1a). Subsequently, the rats were acclimated in the CRS induction room for 1 h to habituate to the environment before starting the behavioral experiments. The first CRS session was conducted after all baseline behavioral tests were completed. The animals were re-examined for their behaviors on days 7, 14, and 28. The following behavioral tests were used: FST, SPT, and open-field test (OFT), with one test conducted per day in that order (Figure 1b). After the final behavioral test, the rats were sacrificed and their brains were removed to determine dendritic spine density and hippocampal synaptic function by Golgi–Cox staining and hippocampal LTP recording, respectively (Figure 1b).

### Drugs and treatments

CBD (Love Hemp, London, UK), provided by the MAUMANDSON Co., Ltd. (Bangkok, Thailand), was dissolved in a vehicle solution comprising 0.9% normal saline, 4% dimethyl sulfoxide (DMSO; Sigma-Aldrich, St. Louis, MO, USA), and 2% polysorbate 80 (Tween 80; Sigma-Aldrich, Steinheim, Germany) to prepare final concentrations of 10, 30, and 100 mg/kg [30]. All CBD was obtained from the same batch (Batch no. TST-2246) with 99.73% purity. The test concentration (10 mg/kg) of escitalopram (Lexapro; H. Lundbeck A/S, Australia) was prepared from a stock solution of 20 mg/mL. The vehicle group received only the vehicle solution (4% DMSO and 2% Tween 80 in 0.9% normal saline). All drugs were intraperitoneally injected daily into the rats 1 h before restraint stress.

### CRS protocol

The rats were immobilized daily in a restrainer, a plastic tube sized to fit the body size of each rat, for 3 h/day (9.00–12.00 a.m.) for 28 days. This procedure was conducted in a room with restricted access.

### Behavioral tests

#### OFT

The OFT is used to determine rodents’ locomotor activities [31]. Rats were acclimated in the testing room for 1 h before starting OFT. Rats were placed in a square open-field arena (100 × 100 cm) surrounded by a 50 cm–high wall. Each rat was placed at the center of the arena, allowing them to explore the arena for 10 min. Rat behavior was recorded using a video camera and analyzed using the Panlab SMART video tracking system version 3.0 (Panlab, Barcelona, Spain). The total distance traveled was used to measure rat locomotion.

#### FST

FST is commonly used to assess depression-like behaviors in rodents [32]. The rats were acclimated in the testing room for 1 h before starting the FST. Rats were placed in a transparent cylindrical tank (diameter: 30 cm; height: 50 cm) filled with water maintained at 25°C to a depth of 30 cm. The FST comprised training and testing sessions. In the training session, conducted 24 h before testing, rats swam for 15 min without recording. On the testing day, the rats swam for 5 min while their behavior was recorded. The immobility time (floating duration) was analyzed. The videos were analyzed using the Panlab SMART video tracking system. Rats generally attempt to escape the water; therefore, increased time spent floating indicates behavioral despair.

#### SPT

The SPT is used to screen for behaviors that indicate anhedonia, another core symptom of depression [33]. Briefly, the rats were deprived of food and water for 21 h. Subsequently, they were given free access to 1% sucrose solution and tap water for 3 h (test duration, with no food in the cage). The volumes of the sucrose solution and water were then measured. Sucrose preference was calculated as the percentage of 1% sucrose solution consumed relative to total liquid intake using the following equation:

Sucrose preference (%) = [1% sucrose solution intake (mL)/total liquid intake (mL)] × 100.

Rats typically prefer sweet water or sucrose solution over tap water. Therefore, a reduced preference for sweetened water indicates anhedonia.

### Hippocampal LTP

The rats were deeply anesthetized with isoflurane and then decapitated. Brains were extracted and immersed in oxygenated artificial cerebrospinal fluid (ACSF) (components: 124 mM NaCl, 3 mM KCl, 1.25 mM NaH_2_PO_4_-2H_2_O, 1.30 mM MgSO_4_-7H_2_O, 26 mM NaHCO_3_, 10 mM D-glucose, and 2.4 mM CaCl_2_-2H_2_O) at 4°C for 3 min. Using a vibratome (Leica Biosystems, Germany), the brain was sectioned into 400 μm–thick transverse hippocampal slices in the coronal plane. These sections were further divided into the right and left halves. Brain sections were incubated in a tissue chamber with oxygenated ACSF at room temperature (25°C) for at least 1 h before electrophysiological recording.

The strength of hippocampal Cornu Ammonis 3-Cornu Ammonis 1 (CA1) synapses was assessed through extracellular recordings at hippocampal sites using a light microscope (Olympus, Japan) with a 3–5 MΩ capillary recording electrode filled with ACSF. The electrode was precisely positioned in the dendritic layer of the CA1 region to record field excitatory postsynaptic potentials (fEPSPs). Simultaneously, a stimulating electrode was placed 1,000 μm away within the same dendritic layer to activate the Schaffer collateral-commissural fibers. The fEPSPs were recorded once every 30 s using the Lab Chart 7 program (AD Instruments, Australia) at a constant single shock stimulation current.

The I–O curve of each brain slice was recorded by gradually increasing the stimulus intensity from 30 μA to 300 μA to determine basal synaptic transmission. The stimulus intensity that elicited 40%–50% of the maximum fEPSP amplitude was maintained throughout the experiment on each hippocampal slice. The baseline activity of each hippocampal slice was recorded for 15 min, and those with an unstable baseline recording were excluded. LTP was induced by applying high-frequency stimulation (100 Hz and 100 pulses) twice at the same stimulus intensity. We continuously recorded fEPSPs for 120 min to assess the LTP magnitude, and the peak slope of the rising phase of the fEPSPs (1 ms duration) was calculated [34].

### Golgi–Cox staining

Golgi–Cox staining was performed to evaluate the density of the hippocampal dendritic spine [35]. Briefly, rats were euthanized and immediately perfused with normal saline and 4% paraformaldehyde in 0.1 M phosphate buffer at pH 7.4. Rat brains were extracted and immersed in 4% paraformaldehyde for 24 h. Subsequently, the brains were immersed in the Golgi–Cox solution and stored in the dark for 2 days at 25°C. The old Golgi–Cox solution was replaced with a fresh one and stored in the dark at 25°C for 12 days. Following Golgi–Cox solution immersion, the solution was replaced with 30% sucrose solution and the brains were refrigerated until they sank, taking approximately 2 weeks.

Using a vibratome, the brains were sliced into coronal sections at 100–200 μm thickness. Brain sections were placed on 0.5% gelatin-coated slides and stored at 25°C overnight. The staining procedure began with two 1-min washes of the sections in distilled deionized (DDI) water. Subsequently, the sections were immersed in 30% ammonium hydroxide solution at 25°C for 30 min in the dark, followed by washing twice with DDI water (1 min/wash). Subsequently, the sections were treated with a 15% Kodak Fixer solution at 25°C for 10 min in the dark and washed with DDI water. Next, they were serially dehydrated for 1 min each with graded alcohol solutions, including 50%, 70%, and 95% ethanol, followed by immersion in 100% ethanol for 15 min. The slide background was cleaned with xylene for an additional 10 min. Next, we immersed the sections in chloroform + xylene + ethanol alcohol solution (chloroform: xylene:ethanol ratio at 1:1:1) for 15 min in the dark. Finally, the slides were mounted with Permount-coated coverslips and left to dry overnight in a fume hood before being examined under a light microscope (Nikon, Japan) [35].

For each section, we captured reference pictures at 5× magnification. Individual neurons and dendritic spines were imaged at 20× and 40× magnification, respectively. A 40× magnification image was used to image ten neurons from each brain section, with five neurons from the right hemisphere and the rest from the left. Either primary or secondary dendritic projections were selected for the analysis. The number of dendritic spines was counted for 10 μm length of each projection, and at least, three neuron projections were collected. The data collected from five sections of each brain were analyzed. Dendritic spine density was manually quantified from images acquired at 40× magnification. Images were analyzed using ImageJ, with manually marked spines and the total number displayed.

### Statistical analysis

All results are presented as means ± standard error of the mean. Data from the behavioral tests were analyzed using two-way repeated-measures analysis of variance (ANOVA) with one between-group factor (treatment) and one within-group factor (time), followed by the least significant difference (LSD) *post hoc* test. We used one-way ANOVA followed by the LSD *post hoc* test for hippocampal dendritic spine density. The fEPSP slopes of LTP were also analyzed using two-way ANOVA, followed by the LSD *post hoc* test. All statistical analyses and graph plotting were performed using GraphPad Prism version 5 (GraphPad by Dotmatics, Boston, USA). An alpha level of 0.05 was used to determine statistical significance.

## RESULTS

### Effect of CBD administration on the locomotor activity of OFT rats

**Figure 2:**
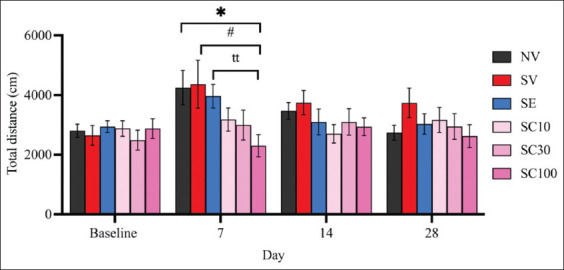
Effect of cannabidiol administration on the locomotor activity of rats in the open-field test. Data are expressed as mean ± standard error of the mean, n = 10 per group. *p < 0.05 compared with the vehicle-treated non-stressed animals. ^tt^p < 0.01 compared with the escitalopram (10 mg/kg)-treated animals with chronic restraint stress. ^#^p < 0.05 compared with the vehicle-treated CRS animals. SC10 group, cannabidiol (CBD, 10 mg/kg)-treated CRS animals; SC30 group, CBD (30 mg/kg)-treated CRS animals; and SC100 group, CBD (100 mg/kg)-treated CRS animals. CRS=Chronic restraint stress.

The OFT results revealed no significant differences in locomotor activity between the non-stressed group and the vehicle-treated CRS group at any observed time point (Figure 2), suggesting that CRS did not impair overall locomotor function. On day 28, neither CBD at any dose nor escitalopram produced significant changes in locomotor activity in rats (SV: 3738.36 ± 500.60 vs. SC10: 3166.18 ± 418.29, F[5, 54] = 0.8771, p = 0.3923; vs. SC30: 2946.12 ± 428.78, F[5, 54] = 1.202, p = 0.2453; vs. SC100: 2622.95 ± 383.35, F[5, 54] = 1.769, p = 0.0950; vs. SE: 3032.056 ± 340.57, F[5, 54] = 1.167, p = 0.2606).

### CBD attenuated depression-like behaviors

#### Effect of CBD on CRS-exposed FST rats

**Figure 3:**
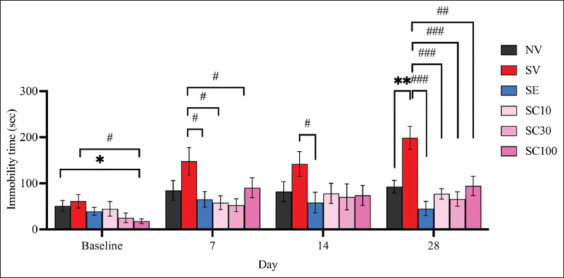
Cannabidiol may reverse behavioral despair in rats subjected to chronic restraint stress, as measured by the forced swim test. Data are expressed as means ± standard error of the mean, n = 10 per group. *p < 0.05 and **p < 0.01 compared with the vehicle-treated non-stressed animals. ^#^p < 0.05, ^##^p < 0.01, and ^###^p < 0.001 compared with the vehicle-treated animals with SV. SE group, escitalopram (10 mg/kg)-treated CRS animals; SC10 group, cannabidiol (CBD, 10 mg/kg)-treated CRS animals; SC30 group, CBD (30 mg/kg)-treated CRS animals; and SC100 group, CBD (100 mg/kg)-treated CRS animals. CRS=Chronic restraint stress, SV=CRS vehicle.

The CRS group exposed for 28 days displayed a significant increase in immobility time in the FST compared with the non-stressed group (Figure 3; NV: 92.9 ± 13.47; SV: 198.7 ± 24.98; F[5, 54] = 3.729, p < 0.01), confirming the successful development of behavioral despair in CRS-exposed rats. On day 7, all CBD groups showed significant antidepressant-like effects, which diminished by day 14 but re-emerged thereafter. On day 28, all CBD doses (10, 30, and 100 mg/kg) significantly reduced immobility time in CRS-exposed rats (Figure 3). Immobility times were as follows: SV: 198.7 ± 24.98 versus SC10: 77.3 ± 11.28, F(5, 54) = 4.430, p < 0.001; versus SC30: 65.7 ± 15.75, F(5, 54) = 4.504, p < 0.001; versus SC100: 94.3 ± 20.76, F(5, 54) = 3.214, p < 0.01. Escitalopram also significantly reduced immobility as early as day 7 (SV: 148.30 ± 29.71 vs. SE: 65.3 ± 16.92, F[5, 54] = 2.428, p < 0.05), with the effect becoming stronger by day 28 (Figure 3; SV: 198.7 ± 24.98 vs. SE: 45.4 ± 15.63, F[5, 54] = 5.203, p < 0.001). By day 28, there were no significant differences between escitalopram and CBD-treated groups (SE: 45.4 ± 15.63 vs. SC10: 77.3 ± 11.28, F[5,54] = 1.655, p = 0.1169; vs. SC30: 65.7 ± 15.75, F[5, 54] = 0.9150, p = 0.3723; vs. SC100: 94.3 ± 20.76, F[5, 54] = 1.882, p = 0.0774). These findings suggest that all CBD doses were comparable to escitalopram in reducing behavioral despair, although the onset of CBD’s antidepressant-like effects may be slower and less sustained.

#### Effect of CBD administration on rats exposed to CRS in the SPT

**Figure 4:**
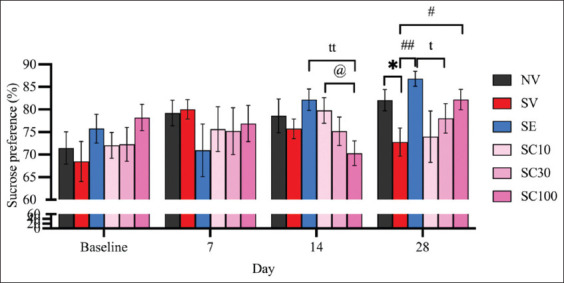
Cannabidiol may reverse anhedonia in rats subjected to chronic restraint stress, as measured by the sucrose preference test. Data are expressed as mean ± standard error of the mean, n = 10 per group). *p < 0.05 compared with the vehicle-treated non-stressed animals. ^t^p < 0.05 and ^tt^p < 0.01 compared with escitalopram (10 mg/kg)-treated animals with chronic restraint stress. ^#^p < 0.05 and ^##^p < 0.01 compared with the vehicle-treated CRS animals. ^@^p < 0.05 compared with cannabidiol (CBD, 10 mg/kg)-treated CRS animals. CBD (30 mg/kg)-treated CRS animals; CBD (100 mg/kg)-treated CRS animals in the SC30 group; CBD (100 mg/kg)-treated CRS animals in the SC100 group. CRS=Chronic restraint stress.

On day 28, sucrose preference was significantly lower in CRS-exposed rats (SV) compared to the non-stressed group (Figure 4; NV: 82.05 ± 2.37 vs. SV: 72.77 ± 3.10, F[5, 54] = 2.378, p < 0.05), confirming the induction of anhedonia. On day 14, neither escitalopram nor any CBD dose reversed anhedonia in CRS rats (SV: 75.71 ± 2.17 vs. SE: 82.17 ± 2.37, F[5, 54] = 2.009, p = 0.0599; vs. SC10: 79.78 ± 2.82, F[5, 54] = 1.144, p = 0.2686; vs. SC30: 75.16 ± 3.16, F[5, 54] = 0.1435, p = 0.8877; vs. SC100: 70.29 ± 2.74, F[5, 54] = 1.550, p = 0.1395). Notably, CBD at 100 mg/kg was significantly less effective than escitalopram at this time point (SE: 82.17 ± 2.37 vs. SC100: 70.29 ± 2.74; F[5,54] = 3.274, p < 0.01). However, by day 28, both escitalopram and CBD at 100 mg/kg reversed anhedonia (Figure 4; SV: 72.77 ± 3.10 vs. SC100: 82.19 ± 2.26, F[5, 54] = 2.455, p < 0.05; vs. SE: 86.79 ± 1.65, F[5, 54] = 3.988, p < 0.01), with no significant differences between them, suggesting that CBD at 100 mg/kg may be as effective as escitalopram in alleviating anhedonia by day 28.

#### Effect of CBD administration on hippocampal LTP

**Figure 5:**
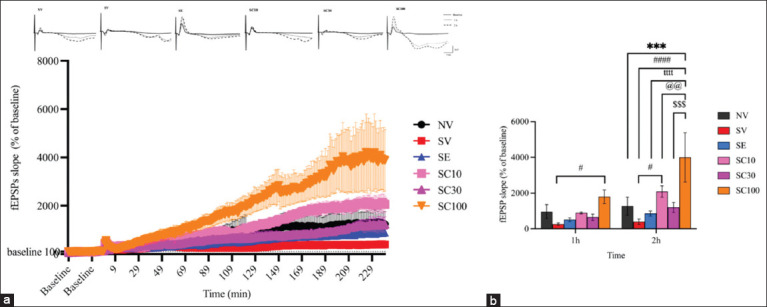
Cannabidiol improved hippocampal long-term potentiation throughout the 2-h period after HFS. (a) Dotted lines represent the percentage of baseline levels recorded before and after HFS over a 2-h period. (b) Bar graph shows the mean percentage of field excitatory postsynaptic potential slopes during the final 10 min of each time point. Data are expressed as mean ± standard error of the mean, n = 4 per group. NV group, vehicle-treated non-stressed animals; SV group, vehicle-treated animals with CRS; SE group, escitalopram-treated CRS animals; SC10 group, cannabidiol (CBD, 10 mg/kg)-treated CRS animals; SC30 group, CBD (30 mg/kg)-treated CRS animals; SC100 group, CBD (100 mg/kg)-treated CRS animals. ***p < 0.001 compared with the vehicle-treated nonstressed animals (NV). ^#^p < 0.05 and ^Vetworld-18-2823-g004.jpg^p < 0.0001 compared with the vehicle-treated animals with CRS (SV). ^tttt^p < 0.0001 compared with escitalopram (10 mg/kg)-treated CRS animals (SE). ^@@^p < 0.01 compared with CBD (10 mg/kg)-treated CRS animals (SC10). ^$$$^p < 0.001 compared with CBD (30 mg/kg)-treated CRS animals (SC30). HFS=High-frequency stimulation, CRS=Chronic restraint stress.

At the 1^st^ h of LTP recording, no significant difference in fEPSP slopes was observed between NV and SV groups (Figures 5a and 5b; NV: 963.40 ± 390.4 vs. SV: 261.63 ± 68.13, F[5, 18] = 1.041, p = 0.3049). However, the SC100 group demonstrated significant LTP enhancement compared with the SV group (Figures 5a and 5b; SV: 261.63 ± 68.13 vs. SC100: 1796.47 ± 381.86, F[5, 18] = 2.276, p < 0.05). Other CBD doses did not show significant changes relative to SV (SC10: 887.54 ± 54.28, F[5, 18] = 0.9283, p = 0.3594; SC30: 647.42 ± 176.92, F[5, 18] = 0.5722, p = 0.5707). At the 2^nd^ hour, NV and SV groups again showed no significant differences (NV: 1270.35 ± 501.32 vs. SV: 395.00 ± 150.24, F[5, 18] = 1.298, p = 0.2025). Yet, the SC100 group showed markedly higher fEPSP slopes compared with NV (1270.34 ± 501.32 vs. 3995.54 ± 1380.02, F[5, 18] = 4.042, p < 0.001). Moreover, SC10 and SC100 groups displayed significant increases compared with SV (SC10: 2082.34 ± 321.77, F[5, 18] = 2.503, p < 0.05; SC100: 3995.54 ± 1380.02, F[5, 18] = 5.340, p < 0.0001). Thus, CBD at 10 and 100 mg/kg enhanced hippocampal synaptic strength, which may contribute to reducing depression-like behaviors in CRS rats. In contrast, escitalopram did not enhance LTP magnitude, suggesting different molecular effects compared with CBD.

#### Effect of CBD administration on hippocampal dendritic spine density

**Figure 6:**
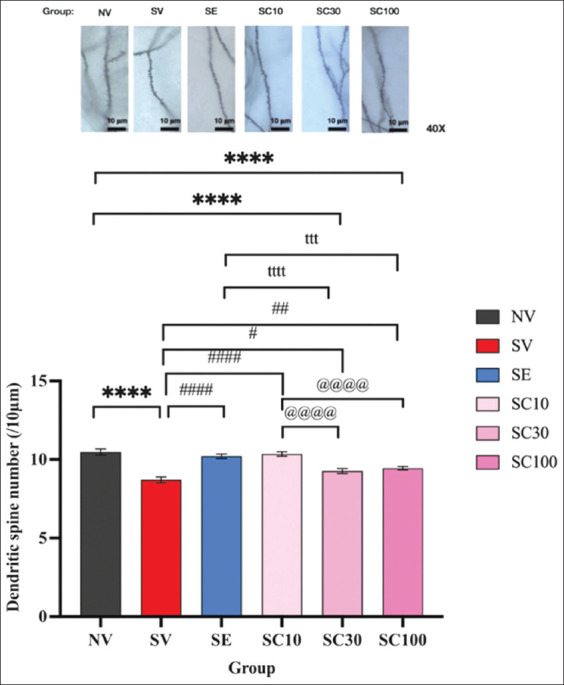
Cannabidiol treatment improved hippocampal dendritic spine density. Data are expressed as mean ± standard error of the mean, n = 4 rats per group. ****p < 0.0001 compared with the vehicle-treated non-stressed animals. ^#^p < 0.05, ^##^ p < 0.01, and ^Vetworld-18-2823-g006.jpg^p < 0.0001 compared with the vehicle-treated animals with CRS. ^ttt^p < 0.001 and ^tttt^p < 0.0001 compared with escitalopram-treated CRS animals. ^@@@@^p < 0.0001 compared with cannabidiol (CBD, 10 mg/kg)-treated CRS animals. CBD (30 mg/kg)-treated CRS animals in the SC30 group; CBD (100 mg/kg)-treated CRS animals in the SC100 group. CRS=Chronic restraint stress.

CRS significantly reduced hippocampal dendritic spine density after 28 days (Figure 6; NV: 10.48 ± 0.19 vs. SV: 8.70 ± 0.18, F[5, 234] = 7.955, p < 0.0001). Conversely, all CBD doses and escitalopram significantly increased dendritic spine density compared with SV (SC10: 10.35 ± 0.14, F[5, 234] = 7.406, p < 0.0001; SC30: 9.27 ± 0.16, F[5, 234] = 2.532, p < 0.05; SC100: 9.45 ± 0.11, F[5, 234] = 3.328, p < 0.01; SE: 10.21 ± 0.14, F[5, 234] = 6.745, p < 0.0001). Notably, CBD at 10 mg/kg significantly outperformed CBD at 30 mg/kg (10.35 ± 0.14 vs. 9.27 ± 0.16, F[5, 234] = 4.874, p < 0.0001) and 100 mg/kg (vs. 9.45 ± 0.11, F[5, 234] = 4.078, p < 0.0001). Similarly, escitalopram significantly outperformed CBD at 30 mg/kg (10.21 ± 0.14 vs. 9.27 ± 0.16, F[5, 234] = 4.213, p < 0.0001) and 100 mg/kg (vs. 9.45 ± 0.11, F[5, 234] = 3.417, p < 0.001), though no significant difference was found between SC10 and SE groups. These findings suggest that CBD exerts an inverse dose-dependent effect on dendritic spine density, where a lower dose enhances hippocampal neuroplasticity more effectively.

## DISCUSSION

### Depression and its core symptoms

Depression is a serious mental disorder with a substantial global burden [1] and an increased risk of suicide [36]. Its core symptoms are anhedonia (the inability to experience pleasure) and despair (the complete loss or absence of hope, representing a depressed mood) [37, 38]. The FST and SPT are well-known behavioral tests used in preclinical research to study depression-like behaviors in animal models. Despair or “behavioral despair” is measured using the FST, whereas anhedonia is measured using the SPT. Behavioral despair and anhedonia are considered behavioral analogs of core depressive symptoms and are commonly modeled in preclinical studies of depression [39].

### Role of chronic stress in depression

Chronic stress is a major risk factor for depression. One of its primary mechanisms involves the dysregulation of the hypothalamic–pituitary–adrenal axis, causing the sustained release of glucocorticoids, such as cortisol [40]. Prolonged exposure to high glucocorticoid levels causes significant hippocampal changes, including reduced BDNF levels, decreased dendritic spine density, and impaired LTP [22, 23]. These changes result in reduced synaptic plasticity, neuronal atrophy, and disrupted neural connectivity, all of which are linked to the cognitive and emotional symptoms of depression [22, 24].

In this study, depression was successfully induced in a rat model using CRS for 3 h/day for 28 days. The SV group exhibited increased immobility duration in the FST and decreased sucrose preference in the SPT, indicating behavioral despair and anhedonia measured by the FST and SPT, respectively. These behavioral changes were unlikely due to impaired locomotion, as no significant difference was observed between the SV and NV groups in the OFT. Moreover, the dendritic spine density was significantly reduced in CRS-exposed animals in the SV group compared with that in non-stressed animals in the NV group. The reduction in dendritic spine density might be caused by decreased BDNF expression [41] and prolonged glucocorticoid exposure [42], leading to impaired synaptic plasticity and reduced synaptic connectivity in the hippocampus, eventually contributing to depression [43–45]. These results emphasize the effect of chronic stress on the hippocampus and the successful development of depression in our model.

### Current treatments for depression

Treatment for depression often involves psychotherapy, medication, neuromodulation, and lifestyle changes. Antidepressant medications are commonly prescribed to manage depressive symptoms. Escitalopram, an SSRI, is widely used in patients with MDD [46]. It prevents serotonin transporters from reuptaking serotonin into the presynaptic membrane, thereby increasing serotonin levels in the synapses. Serotonin acts on serotonin receptors, which are present in various brain regions, particularly in the hippocampus. Serotonin receptor activation can influence synaptic plasticity [47], which is the ability of synapses to strengthen or weaken over time, depending on their activities [47, 48].

Dysfunction in hippocampal neuroplasticity, including reduced neurogenesis, dendritic atrophy, and impaired synaptic plasticity, disrupts the neural circuits involved in mood regulation and cognitive functions, contributing to the pathophysiology of depression [11]. As expected, the SE group showed reduced immobility time in FST, increased sucrose preference in SPT, and increased dendritic spine density compared with those of the SV group. Nonetheless, patients with MDD taking escitalopram or other SSRIs exhibit adverse effects, such as nausea, diarrhea, headache, and insomnia [49]. Thus, molecules with different antidepressant action mechanisms and better tolerability profiles than SSRIs may help relieve depressive symptoms and improve patients’ quality of life.

### CBD as a potential antidepressant

CBD has been widely used for therapeutic and medical purposes in many countries, such as the United States, Canada, Australia, and New Zealand [50], and it appears to be safe, with common side effects typically being mild and transient (e.g., decreased appetite, diarrhea, and somnolence) [13]. CBD possesses many therapeutic effects, such as antidepressant, anxiolytic, anticonvulsive, antipsychotic, and neuroprotective effects [13]. Although the antidepressant effect of CBD has been demonstrated [13, 20], its effect on neuroplasticity in the hippocampus, a critical brain structure involved in depression, remains unclear. Therefore, this study aimed to investigate the antidepressant-like effect of CBD and its underlying mechanisms in the hippocampus, particularly in terms of dendritic spine density and LTP.

### Effects of CBD on depression-like behaviors

In the present study, CBD, particularly at 100 mg/kg dose, alleviated depression-like behaviors in stressed animals (SV group) by increasing the sucrose preference (anti-anhedonia) and decreasing the immobility time (anti-despair) compared with that of vehicle treatment. These results are consistent with those of previous studies [17, 26, 27] demonstrating that CBD administration mitigates depression-like behaviors induced by CRS in rats. Moreover, the antidepressant effects of CBD (100 mg/kg) were as effective as those of escitalopram, considering that no significant difference was observed between the SE and SC100 groups. Thus, CBD at its optimal dose may have non-inferior potency to escitalopram.

### CBD and synaptic plasticity

CBD also increased hippocampal dendritic spine density in the SC10, SC30, and SC100 groups. The increase in hippocampal spine density could be associated with the therapeutic action of CBD on CRS-induced depression-like behaviors in these rats. Thus, the antidepressant-like effect of CBD may improve dendritic spine density in the hippocampus. Notably, the inverse dose-dependent effect of CBD on dendritic spine density enhancement (Figure 6), with higher doses promoting less neuroplasticity in the hippocampus, may be attributed to the sedative effects of higher CBD doses, which can impair learning [28, 29].

Hippocampal LTP is a process related to synaptic strengthening, which is crucial for learning and memory [51]. Depression is associated with impaired synaptic plasticity, particularly in the hippocampus and prefrontal cortex, which are brain regions involved in mood regulation, cognition, and stress response [52]. Reduced LTP has been observed in animal models of chronic stress and depression, correlating with cognitive deficits and anhedonia [45, 53]. LTP enhancement by antidepressants indicates improved neuronal communication, neural circuit remodeling, and potential relief from depressive symptoms [54, 55]. However, CBD at 100 mg/kg, but not escitalopram, substantially enhanced hippocampal LTP magnitude compared with the NV, SV, and SE groups. Thus, CBD may enhance hippocampal synaptic strength and plasticity, potentially alleviating depression.

### Mechanistic insights: Endocannabinoid and BDNF pathways

CBD can modulate the endocannabinoid system in the brain by inhibiting the fatty acid amide hydrolase (FAAH) enzyme, which metabolizes anandamide, primarily arachidonoyl ethanolamide (AEA) [25, 56]. This inhibition increases AEA levels in the brain. The AEA then activates CB receptors, leading to neurotransmission modulation [25, 57, 58]. An imbalance between the primary excitatory neurotransmitter (glutamate) and inhibitory neurotransmitter (gamma-aminobutyric acid [GABA]) systems in the brain has been linked to MDD development [25]. This imbalance can influence the overall tone of the hippocampal network [59], which plays a critical role in mood regulation [59].

AEA activates CB1 receptors on both GABAergic and glutamatergic neurons in the hippocampus, reducing the release of GABA and glutamate by inhibiting calcium influx [60]. This process decreases inhibitory control and dampens excitatory signaling, thereby helping maintain a balanced neural activity. Thus, AEA can regulate glutamate and GABA release through the CB1 receptor [25]. Furthermore, the administration of FAAH inhibitor (URB597) leads to AEA accumulation in the rat’s hippocampus [61]. Repeated administration of URB597 increases serotonin release in the hippocampus [61].

In addition, by restoring reduced glutathione and increased lipid hydroperoxide levels, along with decreased superoxide dismutase activity observed in the hippocampus and/or prefrontal cortex, AEA normalizes NA levels in the prefrontal cortex and induces a neuroprotective effect [25]. These regions play crucial roles in mood regulation, aggression, impulsivity, and decision-making [25]. This indirect effect of CBD may contribute to its antidepressant-like effects [57].

Moreover, CBD can modulate serotonergic activity by indirectly increasing serotonin, as mentioned above. However, CBD has only a moderate affinity for serotonin 1A (5-HT1a) receptors [14], which are involved in antidepressant and anxiolytic effects as well as cognitive enhancement [20, 26]. Thus, only a higher CBD dose can effectively bind to 5-HT1a receptors, which could explain why significant antidepressant effects were observed only in the highest-dose group (100 mg/kg) in our study.

CBD may also help increase the expression of BDNF and its receptor, TrkB, along with activation of its downstream signaling pathway in the hippocampus and prefrontal cortex [17, 19, 62]. The increase in BDNF/TrkB signaling leads to an increase in hippocampal synaptic plasticity [63, 64], as supported by the findings of this study showing that CBD increased hippocampal dendritic spine density and LTP, along with reductions in depressive symptoms [11]. This mechanism may also underlie the antidepressant effect of CBD, contributing to the alleviation of depressive symptoms [20, 65].

### Comparative efficacy with escitalopram

Although CBD at 100 mg/kg appears to be as effective as escitalopram in treating depression-like behaviors, CBD is more advantageous than escitalopram in enhancing hippocampal synaptic function and plasticity. CBD could substantially enhance hippocampal LTP magnitude, whereas the 10 mg/kg concentration of escitalopram did not. CBD increases the expression of BDNF [17], a crucial protein involved in facilitating LTP in the hippocampus through N-methyl-D-aspartate receptor-dependent mechanisms. By inhibiting FAAH, CBD modulates endocannabinoid tone and increases anandamide levels, thereby regulating presynaptic glutamate release and promoting short- and long-term synaptic plasticity through CB1 receptor signaling [66].

In addition, CBD activates transient receptor potential vanilloid 1, 5-HT1A receptors, and peroxisome proliferator-activated receptor-gamma, all of which influence neuronal excitability and plasticity [67–69]. These mechanisms highlight the direct effects of CBD on glutamatergic plasticity, BDNF expression, and endocannabinoid signaling as key contributors to LTP enhancement. In contrast, escitalopram, an SSRI, increases extracellular serotonin but exerts slower and largely indirect effects on synaptic plasticity [70]. Overall, these findings help explain why CBD enhances LTP, whereas escitalopram does not.

### Clinical translation and relevance

Serotonin is involved in reinforcement learning in healthy people [71]. From a clinical perspective, a substantial number of patients with MDD develop SSRI-associated emotional blunting, a persistent diminution in both positive and negative emotional valence [72] after chronic SSRI use, thereby hampering patients’ quality of life and increasing the risk for medication non-adherence and relapse. One of the probable mechanisms underlying SSRI-associated emotional blunting is lower reinforcement learning [71] and a desensitization of the presynaptic 5HT1a receptor activity [72].

CBD has different mechanisms and, as observed in our study, may help in other hippocampal cognitive functions, such as learning and memory. Accordingly, CBD may be of clinical benefit to patients experiencing SSRI-associated emotional blunting. However, we have not yet investigated this possibility, along with the underlying detailed molecular signaling pathways that may enhance hippocampal dendritic spine density and hippocampal LTP, which must be further studied in subsequent experiments. These findings highlight the potential of CBD as an effective antidepressant-like agent and as a modulator of hippocampal synaptic plasticity, indicating its utility in the development of novel therapeutics for stress-related depression, particularly for patients who are unresponsive to or intolerant of SSRIs. The dose-dependent effects observed in this study may guide future research in optimizing CBD dosing strategies to maximize therapeutic efficacy while minimizing potential adverse effects.

### Limitations

Considering the limitations, the results of this study must be interpreted with caution. First, the sedative effect of CBD can confound the interpretation of animal behaviors in animal models of depression. In FST, rats may spend more time floating due to the sedative effect of CBD than behavioral despair. Conversely, in the early phase of CBD treatment, rats may consume less sucrose in the SPT due to the sedative side effect and may consume more sucrose later due to better tolerability of the sedative side effect or hunger, rather than CBD’s anti-anhedonic effect.

As shown in Figure 4, the sucrose consumption of the CBD-treated rats was inversely related to the CBD dose, possibly due to the dose-dependent sedation of CBD. Subsequently, as this transient side effect subsided, the rats showed increased sucrose consumption, displaying the opposite trend.

Second, translating preclinical findings to clinical settings remains challenging due to the poor oral bioavailability of CBD. CBD undergoes extensive first-pass metabolism, meaning that significantly higher doses are required when administered orally compared with parenteral routes. In this study, we used an intraperitoneal injection, a route known for its higher bioavailability and faster onset of action than oral administration. Nevertheless, a relatively high dose of CBD (100 mg/kg) was still necessary to elicit significant antidepressant-like effects in CRS-exposed rats. Most preclinical studies also use intraperitoneal CBD administration to take advantage of its pharmacokinetic benefits. However, this method differs considerably from the oral route commonly used in human clinical applications. Oral CBD has lower and more variable absorption, contributing to a translational gap that limits the direct applicability of animal research findings to human treatment protocols. To circumvent the limitations associated with oral delivery, specifically, gastrointestinal degradation and first-pass hepatic metabolism, intraperitoneal administration was opted in this study. Nevertheless, future studies should incorporate oral administration in animal models to better approximate human pharmacokinetics and enhance translational relevance.

Third, although high-dose CBD appeared to dramatically enhance LTP magnitude, its effect on hippocampal dendritic spine density was not better than that of escitalopram. Regarding the considerably higher cost of CBD extract than escitalopram and its low bioavailability, CBD might not be a pragmatic option for all patients with MDD. Personalized treatment strategies targeting a specific cohort are crucial in the clinical use of CBD.

Fourth, this study exclusively used male rats to minimize the effects of fluctuating sex hormones on behavioral and physiological responses. Considering the importance of sex differences in neuropsychiatric research, future studies should include both sexes to provide a more comprehensive understanding of the observed effects.

Fifth, future studies should examine additional brain regions, such as the prefrontal cortex and amygdala, to provide a more comprehensive understanding of the neurobiological effects of CBD in depression-related models.

## CONCLUSION

This study demonstrated that CRS successfully induced depression-like behaviors in rats, as evidenced by increased immobility in the FST, reduced sucrose preference in the SPT, and decreased hippocampal dendritic spine density. These findings confirm the role of chronic stress in disrupting hippocampal neuroplasticity and support its use as a valid preclinical model of depression. Importantly, CBD at an optimal dose of 100 mg/kg effectively alleviated both despair- and anhedonia-like behaviors, increased dendritic spine density, and substantially enhanced hippocampal LTP, demonstrating its potential antidepressant-like properties. Comparatively, CBD performed as effectively as escitalopram in reducing depressive behaviors, while showing superior efficacy in strengthening synaptic plasticity.

From a practical perspective, these findings suggest that CBD could serve as a novel therapeutic option for patients with MDD, particularly for those who are unresponsive to or intolerant of SSRIs. Its unique multimodal mechanisms, modulating endocannabinoid tone, enhancing BDNF expression, and influencing serotonergic signaling, highlight its value in improving not only mood-related symptoms but also hippocampal-dependent functions such as cognition, learning, and memory. Moreover, CBD may offer clinical benefits in cases of SSRI-associated emotional blunting, a common limitation of current pharmacotherapies.

The strength of this study lies in its comprehensive evaluation of behavioral, morphological, and electrophysiological outcomes, providing convergent evidence for the antidepressant-like potential of CBD. In addition, the comparison with escitalopram provides clinically relevant context, highlighting CBD’s advantages beyond symptom relief.

Nevertheless, future investigations should address limitations such as the sedative effects of CBD, dose-dependent variability, poor oral bioavailability, and the exclusive use of male rats. Research involving oral administration models, female cohorts, and additional brain regions (e.g., prefrontal cortex and amygdala) will improve translational relevance. Furthermore, clinical studies should optimize dosing regimens and evaluate long-term safety, cost-effectiveness, and potential patient subgroups that may benefit most from CBD therapy.

This study provides compelling preclinical evidence that CBD exerts significant antidepressant-like effects through the enhancement of hippocampal synaptic plasticity. Its multimodal mechanism and favorable tolerability profile position it as a promising candidate for the development of next-generation antidepressants. While further translational and clinical studies are essential, CBD holds potential to complement or even surpass existing treatments, offering new hope in the management of stress-related depression.

## DATA AVAILABILITY

The datasets generated and analyzed during the present study are available from the corresponding author.

## AUTHORS’ CONTRIBUTIONS

NP: Study conception and design, data analysis and interpretation, and drafted and revised the manuscript. JR: Data acquisition, analysis, and interpretation and drafted the manuscript. NT and AV: Data acquisition and analysis. SN, PP, ST, and CC: Study conception and design and revised the manuscript. All authors have read and approved the final manuscript.
